# Analysis of *RAD51D* in Ovarian Cancer Patients and Families with a History of Ovarian or Breast Cancer

**DOI:** 10.1371/journal.pone.0054772

**Published:** 2013-01-25

**Authors:** Ella R. Thompson, Simone M. Rowley, Sarah Sawyer, kConFab, Diana M. Eccles, Alison H. Trainer, Gillian Mitchell, Paul A. James, Ian G. Campbell

**Affiliations:** 1 VBCRC Cancer Genetics Laboratory, Peter MacCallum Cancer Centre, East Melbourne, Victoria, Australia; 2 Familial Cancer Centre, Peter MacCallum Cancer Centre, East Melbourne, Victoria, Australia; 3 Kathleen Cuningham Foundation for Research into Familial Breast Cancer (kConFab), Peter MacCallum Cancer Centre, East Melbourne, Victoria, Australia; 4 Cancer Sciences Division, Faculty of Medicine, University of Southampton, Princess Anne Hospital, Southampton, United Kingdom; 5 Sir Peter MacCallum Department of Oncology, University of Melbourne, Parkville, Victoria, Australia; 6 Department of Pathology, University of Melbourne, Melbourne, Victoria, Australia; University of Illinois at Chicago, United States of America

## Abstract

Mutations in *RAD51D* have been associated with an increased risk of hereditary ovarian cancer and although they have been observed in the context of breast and ovarian cancer families, the association with breast cancer is unclear. The aim of this current study was to validate the reported association of *RAD51D* with ovarian cancer and assess for an association with breast cancer. We screened for *RAD51D* mutations in *BRCA1*/*2* mutation-negative index cases from 1,060 familial breast and/or ovarian cancer families (including 741 affected by breast cancer only) and in 245 unselected ovarian cancer cases. Exons containing novel non-synonymous variants were screened in 466 controls. Two overtly deleterious *RAD51D* mutations were identified among the unselected ovarian cancers cases (0.82%) but none were detected among the 1,060 families. Our data provide additional evidence that *RAD51D* mutations are enriched among ovarian cancer patients, but are extremely rare among familial breast cancer patients.

## Introduction

RAD51 homolog D (S. cerevisiae) (*RAD51D*/*RAD51L3*; MIM#602954) is a component of the homologous recombination DNA repair pathway. The RAD51D protein forms a protein complex with RAD51B, RAD51C and XRCC2 that binds to single stranded DNA (including single stranded gaps in double stranded DNA) and is required for the formation of RAD51 foci in response to DNA damage [1], [2]. Loveday et al [3] recently reported the identification of eight truncating mutations in *RAD51D* among 911 families with histories of breast and ovarian cancer, compared to one mutation among 1,060 population controls. They reported a significantly elevated risk of ovarian cancer (6.30, 95% CI 2.86–13.85) but did not detect a significantly elevated risk of breast cancer (1.32, 95% CI 0.59–2.96). They also reported that mutations are more prevalent in multiple case ovarian cancer families. *RAD51D* has subsequently been investigated in an additional series of 175 breast and ovarian cancer families, with an additional mutation being identified among the 51 families with at least two ovarian cancers (and among the 75 probands affected by ovarian cancer) [4]. Similarly, Pelttari et al [5] identified a splice site mutation (c.576+1G) in two breast cancer affected probands from 95 Finnish breast and/or ovarian cancer families. Pelttari et al then screened for the c.576+1G variant in an additional 2,200 breast and 553 ovarian cancer patients and overall identified 5/707 patients with a personal or family history of ovarian cancer compared to 2/2,105 breast cancer only patients/families.

Until recently, *BRCA1* and *BRCA2* were the only genes known to confer a considerable risk of ovarian cancer (in conjunction with breast cancer) with two recent studies reporting that 13.3–14.1% of unselected high grade ovarian cancers are accounted for by mutations in one of these two genes [6], [7]. A further small proportion of unselected cases carry mutations in *RAD51C* [8], [9]. Loveday et al [3] estimated that 0.6% of unselected ovarian cancer cases will carry *RAD51D* mutations. To validate the association of *RAD51D* mutations to ovarian cancer and assess if there is any risk for breast cancer risk, we screened all coding exons in germline DNA from an unselected cohort of 245 unselected ovarian cancer patients and *BRCA1*/*2*-unrelated index cases from 1,060 breast and/or ovarian cancer families. Exons containing novel, non-synonymous variants among these cases were screened in a panel of 466 cancer-naive control samples.

## Materials and Methods

The unselected ovarian cancer cohort included 245 individuals with various histological subtypes of ovarian cancer (130 serous, 73 endometrioid, 35 mucinous, two clear cell, two granulosa cell tumours, two adenocarcinomas and one mixed mullerian tumour). These samples were obtained from patients presenting to hospitals in the south of England, UK [10]. Undocumented, verbal consent was obtained from patients as approved by the governing ethics committee at the time.

The familial cohort included 540 individuals with verified personal and family histories of breast and/or ovarian cancer who were previously assessed at the Peter MacCallum Cancer Centre Familial Cancer Centre (Australia), as well as index cases from 520 multiple case breast cancer families (with or without ovarian cancer) obtained from the Kathleen Cunningham Foundation Consortium for Research into Familial Breast Cancer (kConFab) [11]. kConFab families are recruited through Familial Cancer Centres throughout Australia and New Zealand. All families were recruited based on multiple affected, mutigenerational family and personal history of breast and/or ovarian cancer. The families fulfilled diagnostic criteria for BRCA testing, with no underlying *BRCA1* or *BRCA2* mutation having been identified. The ethnicity of the index cases was self-reported as Caucasian in the vast majority of cases. All individuals provided written, informed consent for genetic testing of the genetic causes of hereditary breast and ovarian cancer and subsequently tested negative for mutations in *BRCA1* and *BRCA2*. This study was approved by the Peter MacCallum Cancer Centre Human Research Ethics Committee. In total, index cases from 1,060 families were examined in this study, including 16 with a family history of ovarian cancer only, and 303 with a family history of both breast and ovarian cancer. Of these index cases, 98 had a personal history of ovarian cancer. The remaining 741 families had a personal and family history of breast cancer only.

Cancer-naive control DNA samples were obtained from kConFab (231 age- and ethnicity-matched best friend controls) and from the Princess Anne Hospital, UK (235 Caucasian female volunteers, as described previously) [12]. kConFab control individuals provided written, informed consent. Controls from the Princess Anne Hospital provided undocumented, verbal consent as approved by the governing ethics committee at the time.

DNA for mutation screening underwent whole genome amplification (WGA) using the Repli-G amplification system (Qiagen). Ten primer pairs were designed to amplify the ten coding exons of *RAD51D* with amplicons ranging in size from 215–277 bp for high resolution melt (HRM) analysis (Table 1). HRM analysis and DNA resequencing were performed as described previously [13]. Variant positions were determined with reference to GenBank reference sequence NM_002878.3. Nucleotide numbering reflects cDNA numbering with +1 corresponding to the A of the ATG translation initiation codon in the reference sequence, according to HGVS guidelines (www.hgvs.org/mutnomen). All novel variants were verified by Sanger resequencing of non-WGA DNA. Tumour cells were needle dissected from 10 µm sections to obtain tumour DNA, which was subsequently whole genome amplified.

**Table 1:** Primers used for mutation analysis of *RAD51D*.

Primer pair	Forward primer (5′-3′)	Reverse primer (5′-3′)	Product size (bp)	Annealing temperature (°C)
*RAD51D*_ex1	CCGCGAATGCCCACGTGA	AGGTATGCCAGGGCAGTG	228	62
*RAD51D*_ex2	GGGTAGAATTGACACCCCATT	CTCCCAAAGTGCTGGGATTA	264	62
*RAD51D*_ex3	GGTGAATGACACCCTGGGA	AGCATCAAAAGCAGAGCTGAG	241	62
*RAD51D*_ex4	CAGAACCAGTGCTTGAAAGAAA	CCCTGGGCTATGCATCTACC	248	62
*RAD51D*_ex5	GAATCTGGGCAAGGTTTGGT	GGGGTTTTCCTGTGTCAGAA	266	62
*RAD51D*_ex6	TCTTCCTTCTCAGCCTTACC	ATTGCACATCTGCATTTCCA	277	62
*RAD51D*_ex7	TGTGTCCTAGAGGCTGACAGG	GCCAGAGACCAGACTCCAGA	215	62
*RAD51D*_ex8	CCAGCTCTGGAGTCTGGTCT	TTTGGGGTTCAGAAGCTGAC	239	62
*RAD51D*_ex9	CGATGTCCTCTATACTAGCA	CCTCCAGGGCCCAAGATT	261	62
*RAD51D*_ex10	GAGGCTGAAACCTTGCAACT	AGTGCCAGGTGGCAGTAAAC	253	62

The following *in silico* prediction tools were used to assess the likely functional effect of the missense variants identified in this study: PolyPhen-2 [14], SNPs&Go [15], MutPred [16], PMut [17] and MutationTaster [18]. Human Splicing Finder (HSF) was used to assess the effect of all non-truncating variants on splice sites [19].

## Results and Discussion

**Figure 1:**
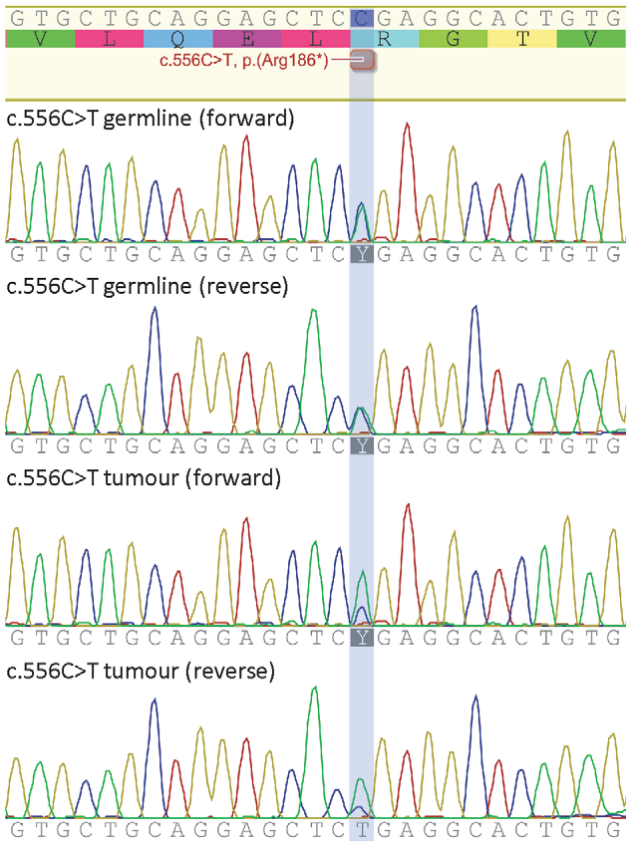
Loss of heterozygosity analysis of the c.556C>T (p.(Arg186*)) variant. Sequencing (forward and reverse) of the heterozygous c.556C>T variant in the germline sample, and tumour DNA showing loss of the wildtype allele (with some contamination from normal DNA).

Two previously reported truncating mutations, p.(Arg186*) and p.(Trp268*) were identified among a series of 245 unselected ovarian cancer patients (0.82%). The p.(Arg186*) variant was detected in a patient diagnosed with a grade 2 papillary serous cystadenocarcinoma at 66 years of age. DNA sequence analysis of tumour tissue obtained from this tumour showed reduction of the wildtype allele consistent with loss of heterozygosity (LOH) (Figure 1). The p.(Trp268*) variant was detected in a patient diagnosed with an endometrioid carcinoma (no grade information) at 70 years of age. No tumour tissue was available for LOH analysis. No family history information is available from either case. The histology of the two ovarian cases with truncating *RAD51D* mutations (*i.e.* high grade serous and endometrioid) is consistent with the majority of mutations reported in other *RAD51D* studies [3], [4], [5], [20], and with other ovarian cancers associated with mutations in double strand break DNA repair genes (*e.g. BRCA1* and *BRCA2*), but the number of mutations in *RAD51D* identified to date is too few to determine the significance of this observation. A third truncating mutation, p.(Lys91Ilefs*13), was identified in one of 466 control samples (0.21%). All three of these mutations have previously been reported [3], [4]. Table 2 provides a summary of all detected variants.

**Table 2:** Summary of germline *RAD51D* variants.

	Site^a^	Nucleotide change^b^^,^ c	Protein change^b^	dbSNP (v135)	Previously described in *RAD51D* mutation studies?	1000 G MAF (%)^d^	EVS MAF (%)^e^	BC families (n = 741)^f^	BC/OC families (n = 303)^f^	OC families (n = 16)^f^	Unselected ovarian cases (n = 245)	Controls (n = 466)^g^	Grantham score^h^	Mutation Taster	MutPred	PMUT	POLYPHEN-2	SNPs&GO
**Truncating**	Ex4	c.270_271dup	p.(Lys91Ilefs*13)	no	Yes [3]	-	-	-	-	-	-	1	-	-	-	-	-	-
	Ex6	c.556C>T	p.(Arg186*)	no	Yes [3], [4]	-	-	-	-	-	1	-	-	-	-	-	-	-
	Ex9	c.803G>A	p.(Trp268*)	no	Yes [3]	-	-	-	-	-	1	-	-	-	-	-	-	-
																		
**Coding non-synonymous**	Ex1	c.47T>C	p.(Met16Thr)	no	-	-	-	1	-	-	-	-	81	Polymorphism	0.587	Neutral	Benign	Neutral
	Ex4	c.286G>T	p.(Gly96Cys)	no	-	-	-	1	-	-	-	-	159	Disease causing	0.938	Pathological	Probably damaging	Disease
	Ex5	c.394G>A	p.(Val132Ile)	rs150498754	-	0.05	0.01	1	-	-	-	N/A	29	Disease causing	0.487	Neutral	Probably damaging	Neutral
	Ex9	c.793G>A	p.(Gly265Arg)	rs140285068	Yes (control) [3], [4]	-	0.02	1	-	-	-	-	125	Disease causing	0.924	Neutral	Probably damaging	Disease
	Ex9	c.796C>T	p.(Arg266Cys)	no	-	-	-	1	-	-	-	-	180	Disease causing	0.621	Pathological	Probably damaging	Neutral
																		
**Coding synonymous**	Ex2	c.117A>T	p.( = )	no	-	-	-	1	-	-	-	N/A	-	-	-	-	-	-
	Ex9	c.864C>T	p.( = )	rs138557828	-	-	0.01	-	-	-	-	1	-	-	-	-	-	-
	Ex9	c.879G>A	p.( = )	no	-	-	0.01	1	-	-	-	-	-	-	-	-	-	-
	Ex9	c.900A>G	p.( = )	no	Yes [3]	-	-	-	-	-	-	1	-	-	-	-	-	-
																		
**Non-coding**	5′UTR	c.-39C>T	p.( = )	no	-	-	-	1	-	-	-	-	-	-	-	-	-	-
	IVS1	c.82+60C>T	p.( = )	no	-	-	-	-	-	-	-	1	-	-	-	-	-	-
	IVS1	c.83-20T>C	p.( = )	rs182793287	-	0.05	-	-	-	-	1	N/A	-	-	-	-	-	-
	IVS3	c.264-6C>T	p.( = )	no	-	-	-	1	-	-	-	-	-	-	-	-	-	-

a Exon (Ex), intervening sequence (IVS).

b Variant positions are reported in reference to NCBI RefSeq NM_002878.3 (mRNA) and NP_002869 (protein).

c In addition to the variants listed, common variants rs4796033, rs28363284 (non-synonymous) and rs9901455 (synonymous) were detected at high frequency in both cases and controls.

d All variants were queried against 1000 Genomes (1000 G) data using the 1000 Genomes Browser which integrates SNP and indel calls from 1,092 individuals (data release 20110521 v3). The minor allele frequency (MAF) is provided here.

e All variants were queried against Exome Variant Server (EVS), NHLBI Exome Sequencing Project (ESP). EVS contains SNP information from 5,379 individuals (data release ESP5400). The minor allele frequency (MAF) is provided here.

f Breast cancer only family (BC), ovarian cancer only family (OC), breast and ovarian cancer family (BC/OC).

g Variants annotated with N/A were not assessed in control samples.

h [22].

Analysis of 1,060 index cases from breast and/or ovarian cancer families did not identify any further truncating mutations. Interestingly, five rare (*i.e.* allele frequency <1%) nonsynonymous variants were detected, once each among 741 breast only cancer families. Three of these variants were novel: p.(Met16Thr), p.(Gly96Cys) and p.(Arg266Cys); the remaining two variants, rs150498754 and rs140285068, are reported in the Exome Variant Server (EVS) database at frequencies of <0.02%. *In silico* analysis tools predicted that variants p.(Gly96Cys) and p.(Arg266Cys) would likely affect protein function (Table 2). Four of eight synonymous or intronic variants detected were novel; these were observed once each in either cases (c.117A>T, c.-39C>T, c.264-6C>T) or controls (c.82+60C>T). None of the synonymous or intronic variants were predicted to alter splicing.

The frequency of truncating germline *RAD51D* mutations detected in all patients with a personal history of ovarian cancer in this study (2/343 = 0.58%) is in keeping with that (0.6%) estimated by Loveday et al., and higher than observed in controls (0.21%). However, it is possible that the variant frequency reported here could be an underestimate due to the reduced sensitivity of HRM analysis compared to direct resequencing (used by Loveday et al.). Five of the variants detected in this study have previously been reported in *RAD51D* mutation studies by Loveday et al. or Osher et al. [3], [4], and may represent founder mutations. However, there is no overlap with variants reported in more recent studies by Pelttari et al. or Wickramanyake et al. [5], [20]. To date, no truncating mutations have been detected among 1,092 individuals in the 1000 genomes cohort (data release 20110521 v3) [21] or 5,379 individuals in the Exome Variant Server (release ESP5400; NHLBI Exome Sequencing Project (ESP), Seattle, WA (http://evs.gs.washington.edu/EVS/) [June 2012]).

The absence of truncating mutations in 741 breast cancer only families (or 962 breast cancer-affected probands) provides further evidence that *RAD51D* mutations do not contribute significantly to breast cancer risk.

## References

[1] Masson J-Y, Tarsounas MC, Stasiak AZ, Stasiak A, Shah R, et al (2001) Identification and purification of two distinct complexes containing the five RAD51 paralogs. Genes & Development 15: 3296–3307. 11751635 10.1101/gad.947001 PMC312846

[2] Smiraldo PG, Gruver AM, Osborn JC, Pittman DL (2005) Extensive Chromosomal Instability in Rad51d-Deficient Mouse Cells. Cancer Research 65: 2089–2096. 15781618 10.1158/0008-5472.CAN-04-2079

[3] Loveday C, Turnbull C, Ramsay E, Hughes D, Ruark E, et al (2011) Germline mutations in RAD51D confer susceptibility to ovarian cancer. Nat Genet 43: 879–882. 21822267 10.1038/ng.893 PMC4845885

[4] Osher DJ, De Leeneer K, Michils G, Hamel N, Tomiak E, et al (2012) Mutation analysis of RAD51D in non-BRCA1/2 ovarian and breast cancer families. Br J Cancer 106: 1460–1463. 22415235 10.1038/bjc.2012.87 PMC3326673

[5] Pelttari LM, Kiiski J, Nurminen R, Kallioniemi A, Schleutker J, et al (2012) A Finnish founder mutation in RAD51D: analysis in breast, ovarian, prostate, and colorectal cancer. J Med Genet 10.1136/jmedgenet-2012-100852 PMC5426530 22652533

[6] Zhang S, Royer R, Li S, McLaughlin JR, Rosen B, et al (2011) Frequencies of BRCA1 and BRCA2 mutations among 1,342 unselected patients with invasive ovarian cancer. Gynecol Oncol 121: 353–357. 21324516 10.1016/j.ygyno.2011.01.020

[7] Alsop K, Fereday S, Meldrum C, deFazio A, Emmanuel C, et al (2012) BRCA Mutation Frequency and Patterns of Treatment Response in BRCA Mutation–Positive Women With Ovarian Cancer: A Report From the Australian Ovarian Cancer Study Group. Journal of Clinical Oncology 10.1200/JCO.2011.39.8545 PMC3413277 22711857

[8] Pelttari LM, Heikkinen T, Thompson D, Kallioniemi A, Schleutker J, et al (2011) RAD51C is a susceptibility gene for ovarian cancer. Hum Mol Genet 20: 3278–3288. 21616938 10.1093/hmg/ddr229

[9] Thompson ER, Boyle SE, Johnson J, Ryland GL, Sawyer S, et al (2011) Analysis of RAD51C Germline Mutations in High-Risk Breast and Ovarian Cancer Families and Ovarian Cancer Patients. Hum Mutat 33: 95. 21990120 10.1002/humu.21625

[10] Bryan EJ, Watson RH, Davis M, Hitchcock A, Foulkes WD, et al (1996) Localization of an ovarian cancer tumor suppressor gene to a 0.5-cM region between D22S284 and CYP2D, on chromosome 22q. Cancer Res 56: 719–721. 8631002

[11] Mann GJ, Thorne H, Balleine RL, Butow PN, Clarke CL, et al (2006) Analysis of cancer risk and BRCA1 and BRCA2 mutation prevalence in the kConFab familial breast cancer resource. Breast Cancer Res 8: R12. 16507150 10.1186/bcr1377 PMC1413975

[12] Baxter SW, Choong DY, Eccles DM, Campbell IG (2002) Transforming growth factor beta receptor 1 polyalanine polymorphism and exon 5 mutation analysis in breast and ovarian cancer. Cancer Epidemiol Biomarkers Prev 11: 211–214. 11867510

[13] Gorringe KL, Choong DY, Williams LH, Ramakrishna M, Sridhar A, et al (2008) Mutation and methylation analysis of the chromodomain-helicase-DNA binding 5 gene in ovarian cancer. Neoplasia 10: 1253–1258. 18953434 10.1593/neo.08718 PMC2570601

[14] Adzhubei IA, Schmidt S, Peshkin L, Ramensky VE, Gerasimova A, et al (2010) A method and server for predicting damaging missense mutations. Nat Methods 7: 248–249. 20354512 10.1038/nmeth0410-248 PMC2855889

[15] Calabrese R, Capriotti E, Fariselli P, Martelli PL, Casadio R (2009) Functional annotations improve the predictive score of human disease-related mutations in proteins. Hum Mutat 30: 1237–1244. 19514061 10.1002/humu.21047

[16] Li B, Krishnan VG, Mort ME, Xin F, Kamati KK, et al (2009) Automated inference of molecular mechanisms of disease from amino acid substitutions. Bioinformatics 25: 2744–2750. 19734154 10.1093/bioinformatics/btp528 PMC3140805

[17] Ferrer-Costa C, Gelpi JL, Zamakola L, Parraga I, de la Cruz X, et al (2005) PMUT: a web-based tool for the annotation of pathological mutations on proteins. Bioinformatics 21: 3176–3178. 15879453 10.1093/bioinformatics/bti486

[18] Schwarz JM, Rodelsperger C, Schuelke M, Seelow D (2010) MutationTaster evaluates disease-causing potential of sequence alterations. Nat Methods 7: 575–576. 20676075 10.1038/nmeth0810-575

[19] Desmet FO, Hamroun D, Lalande M, Collod-Beroud G, Claustres M, et al (2009) Human Splicing Finder: an online bioinformatics tool to predict splicing signals. Nucleic Acids Res 37: e67. 19339519 10.1093/nar/gkp215 PMC2685110

[20] Wickramanyake A, Bernier G, Pennil C, Casadei S, Agnew KJ, et al (2012) Loss of function germline mutations in RAD51D in women with ovarian carcinoma. Gynecol Oncol 127: 552–555. 22986143 10.1016/j.ygyno.2012.09.009 PMC3905744

[21] The 1000 Genomes Project Consortium (2010) A map of human genome variation from population-scale sequencing. Nature 467: 1061–1073. 20981092 10.1038/nature09534 PMC3042601

[22] Grantham R (1974) Amino acid difference formula to help explain protein evolution. Science 185: 862–864. 4843792 10.1126/science.185.4154.862

